# Supplementary figures and images for: Comparative Analysis of Mouse Decidualization Models at the Molecular Level
Source: Genes (Basel). 2020 Aug 13;11(8):935. doi: 10.3390/genes11080935 (PMC7465532; doi:10.3390/genes11080935)

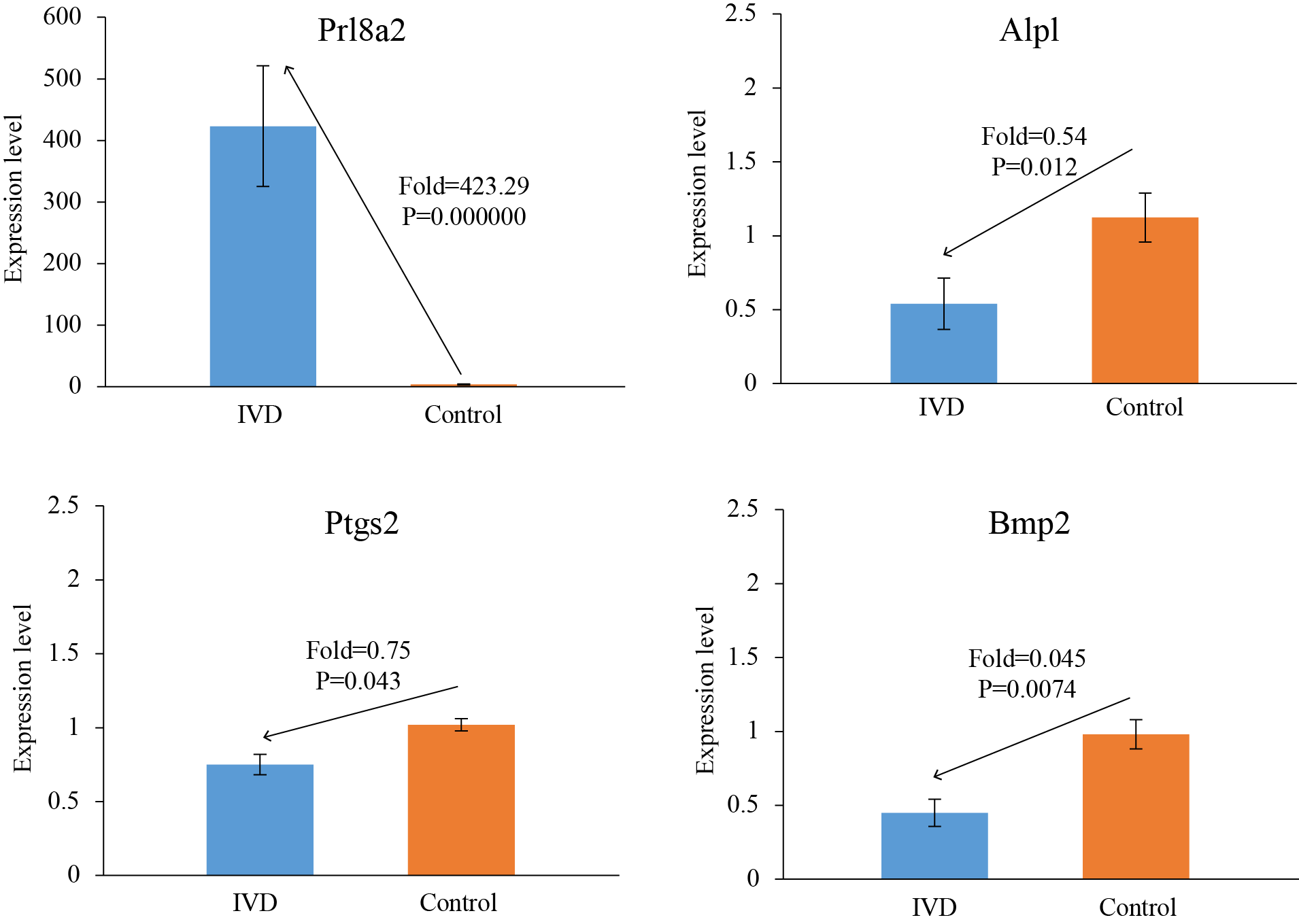

Supplement: Supplementary file 1 [file genes-11-00935-s001.zip › figure-S1.tif]
